# Current Utility of Transgastric Percutaneous Drainage for the Management of Pancreatitis-Related Retrogastric Walled-Off Necrotic Collections: A Prospective Observational Study

**DOI:** 10.7759/cureus.56443

**Published:** 2024-03-19

**Authors:** Sarthak Agrawal, Asmita Arya, Avinash D Gautam, Rajanikant R Yadav, Ashish Singh, Deb Boruah, Samir Mohindra, Archana Gupta, Anshu Srivastava, Mohan Gurjar, Rajneesh K Singh, Rahul Rahul

**Affiliations:** 1 Radiodiagnosis, Sanjay Gandhi Postgraduate Institute of Medical Sciences, Lucknow, IND; 2 Surgical Gastroenterology, Sanjay Gandhi Postgraduate Institute of Medical Sciences, Lucknow, IND; 3 Radiology, All India Institute of Medical Sciences, Guwahati, IND; 4 Gastroenterology, Sanjay Gandhi Postgraduate Institute of Medical Sciences, Lucknow, IND; 5 Pediatric Gastroenterology, Sanjay Gandhi Postgraduate Institute of Medical Sciences, Lucknow, IND; 6 Critical Care Medicine, Sanjay Gandhi Postgraduate Institute of Medical Sciences, Lucknow, IND

**Keywords:** transgastric drainage, percutaneous drainage, walled-off pancreatic necrosis, internalization, acute necrotizing pancreatitis

## Abstract

Introduction

Pancreatic fluid collection (PFC) is one of the most frequent complications associated with acute pancreatitis. The route of drainage is guided by the size and site of collection. The present study aims to assess the clinical and technical success of transgastric percutaneous drainage (PCD) for managing retrogastric walled-off pancreatic necrosis (WOPN).

Materials and methods

A total of 44 patients with acute pancreatitis diagnosed with WOPN who underwent transgastric PCD with ultrasound or CT guidance as part of standard clinical management were included in the study. Patients were observed for improvement in clinical parameters, and treatment outcomes were noted in terms of technical success, clinical success, adverse events, need for additional procedures, hospital stay, and duration of placement of all drains. Data for the internalization of transgastric PCD was also observed in the study.

Results

Technical success during the drain placement was observed in 93% (n=41) of patients.Internalization of the transgastric drain was attempted in 12 patients and successful in 11 (91%). The median duration of hospital stay from the time of placement of the first PCD until discharge and the median duration of all PCDs placed were higher in patients where the transgastric drain was not internalized as compared to patients where the transgastric drain was internalized.

Conclusion

In WOPN, transgastric drain placement and successful internalization in any form help in the early resolution of peripancreatic and abdominal collections. It also reduces the time to percutaneous catheter removal, which in turn reduces the morbidity and decreases the need for additional interventions or surgery.

## Introduction

Pancreatic fluid collection (PFC) is one of the most frequent complications associated with acute pancreatitis. Revised Atlanta classifies PFCs, based on the duration and type of collections, into four distinct subtypes. Acute peripancreatic fluid collections and acute necrotic collections occur within four weeks of disease onset. Beyond four weeks, they may resolve or persist, developing a mature wall to form pseudocysts or walled-off pancreatic necrosis (WOPN), respectively [1]. The majority of the PFCs are asymptomatic and are managed conservatively. Symptomatic PFCs require intervention in the form of drainage [2].

Traditionally, open surgical necrosectomy was the procedure of choice for necrotic collections. It was associated with high morbidity (34-95%) and mortality (6-25%) [2-6]. However, with advancements in imaging and minimally invasive techniques, a step-ladder pattern of management has become the standard of care. It includes percutaneous and endoscopic drainage followed by minimally invasive procedures (laparoscopic drainage, video-assisted retroperitoneal debridement (VARD)), or open necrosectomy as and when required [7-9].

The route of percutaneous drainage (PCD) depends upon the location of the collection. Lesser sac collections can be addressed by the transomental route or perinephric drainage [10]. The associated risk of pancreatic-cutaneous fistulas and infections with these routes can be overcome by endoscopic drainage. An endoscopic ultrasound-guided cystogastric stent can be placed for drainage of retrogastric PFCs [11]. Along with cystogastrostomy, endoscopy-guided transpapillary pancreatic duct stenting can also be done for the management of pancreatic duct disruption [12]. However, endoscopic drainage is not feasible in the following situations: early in the course of the disease when the wall is not formed, collection away from the stomach wall (>1 cm), or if the patient is unable to tolerate the procedure [11]. An image-guided transgastric percutaneous approach can be used for this purpose.

The transgastric percutaneous route has been used since the 1980s to treat pseudocysts. Apart from external drainage and collection in acute situations, the transgastric drain can later be internalized into the stomach lumen, creating a stable cystogastrostomy in ongoing pancreatic fistulae. The role of transgastric PCD in cases of pseudocysts is clearly defined in the literature, but its role in walled-off necrosis patients is a matter of debate.

The present study aims to assess the current utility of transgastric PCD in the management of walled-off lesser sac collections with necrosis.

## Materials and methods

Participants and study design

The study was conducted as a prospective observational study on all patients with walled-off necrotic collections predominantly in the lesser sac regions referred to the department of radiodiagnosis at a tertiary care center.

Inclusion and Exclusion Criteria

All patients with acute necrotizing pancreatitis diagnosed with walled-off necrosis who underwent transgastric PCD under ultrasound or CT guidance as a part of standard clinical management were included in the study.

Patients who refused to give informed consent, individuals who lost follow-up, and cases with contraindication to PCD placement (persistently low platelet count <50,000 cells/ml or deranged international normalized ratio >1.5) were excluded from the study.

After obtaining ethical clearance from the Institute Ethical Committee of Sanjay Gandhi Postgraduate Institute of Medical Sciences (IEC number: 20-26-MD-EXP-35), all the patients fulfilling the inclusion criteria were enrolled.

Image analysis

Imaging of the PFCs was done using ultrasound and CT scans. The following parameters were noted: size of PFCs, number of PFCs, and location. CT severity index, the volume of collection before PCD (ml), and infected collection (if any).

Transgastric PCD

The transgastric percutaneous approach was used for the drainage of PFCs using ultrasound/CT guidance.

Ultrasound-Guided Transgastric Drainage

It was done in patients with large retrogastric fluid collections visible on ultrasonography with the anterior and posterior walls of the collapsed stomach anterior to the collection.

Transgastric drainage was performed using a GE LOGIQ E9 (GE HealthCare, Chicago, Illinois, USA) with a real-time convex transducer. Patients after an overnight fast were positioned supine. Under real-time US guidance, an 18-G CHIBA needle (Cook Medical, Bloomington, Indiana, USA) was inserted percutaneously across the anterior and posterior gastric walls until the collection was entered. A 0.035” Amplatz Ultra Stiff (AUS) guidewire (Cook Medical, Bloomington, Indiana, USA) was passed through the needle into the cyst. The tract was dilated with a 10 or 12 Fr Coons dilator (Cook Medical, Bloomington, Indiana, USA), followed by the insertion of a 10 or 12 Fr multipurpose catheter with a locking loop (Cook Medical, Bloomington, Indiana, USA) over the AUS guidewire. The catheter was fixed to the skin, fluid was aspirated, and it was connected to a urobag.

CT-Guided Transgastric Drainage

CT guidance for placement of drains was used in cases with retrogastric fluid collections not amenable to ultrasound-guided drainage (narrow window, fatty patient).

**Figure 1:**
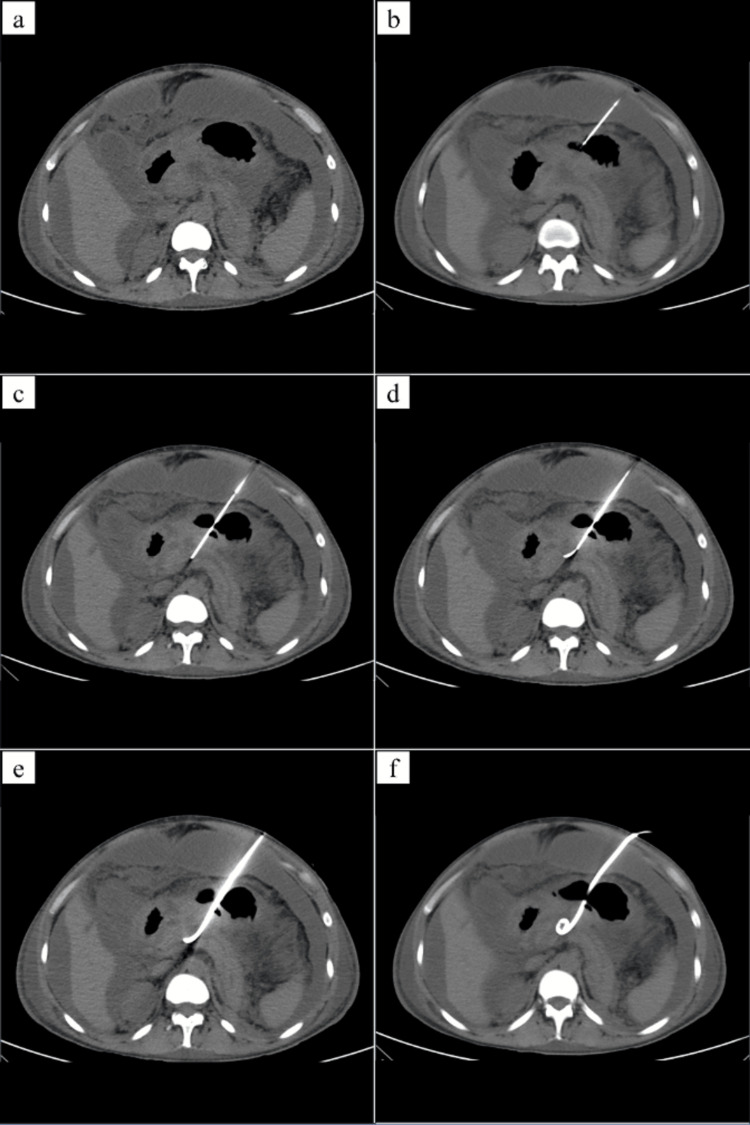
Steps of CT-guided transgastric drain placement (a) An ill-defined, walled-off pancreatic necrosis in the lesser sac. (b) Placement of an 18-G needle across the anterior wall of the stomach. (c) Puncturing both the anterior and posterior walls of the stomach. (d) Passage of 0.038-inch stiff wire through the needle. (e) Dilatation of the tract using 10 Fr Coon’s dilator (f) Placement of 10 Fr locking loop pigtail catheter

As shown in Figure 1, a low mAs (50-100 mAs) non-contrast/contrast-enhanced (in cases where there was substantial vascular structure between the stomach and the retrogastric collection) CT scan of the upper abdomen was done to determine the anatomic relation of the stomach with the retrogastric (lesser sac) PFC on a 64-slice Philips multidetector CT machine (Philips, Amsterdam, Netherlands). Then the stomach was distended with air through a nasogastric tube or by oral effervescent, which contains citric acid, sodium bicarbonate, and sodium carbonate (ENO by GlaxoSmithKline Consumer Healthcare Ltd., India). The skin entry site was selected and marked. Under local anesthesia, an 18-G CHIBA needle was introduced directly into the collection through the anterior abdominal wall and the distended stomach.

The needle position was confirmed, and a 0.035” AUS guidewire was introduced through the CHIBA needle into the collection. The needle was removed, and the tract was dilated using a 10-12 Fr Coon's dilator. Over the guidewire, 10 or 12 Fr multipurpose catheter with a locking loop were placed inside the collection through the dilated tract. A check scan was done to confirm the position, and the catheter was locked and connected to a drainage bag.

Clinical parameters

After placement of the drainage tube, complications related to the procedure and duration of drain placement were observed, and the aspirate was sent for microscopy and culture sensitivity.

Patients were observed daily for changes in clinical parameters. The outcome of the treatment was noted in terms of technical success, clinical success, adverse events (bleeding, perforation, blockage, etc.), need for additional procedures apart from transgastric drainage, total duration of hospital stay, duration of hospital stay from the placement of the first PCD until the removal of the last PCD or internalization of the transgastric PCD, and duration of placement of all PCDs.

Definitions

Clinical improvement was defined by the assessment of the following parameters within seven days after the procedure and compared with pre-procedure values (which were present before the procedure). In the case of spontaneous ventilation, the resolution of either of the following signs/symptoms for 48 hours was noted: fever, vomiting, abdominal pain requiring analgesics, and acceptability of nasogastric/oral feeds.

In the case where the patient was on mechanical ventilation, improvement in either or both of these parameters for 24 hours was observed: a decrease in vasopressor requirement ≥20% from baseline (before the procedure), a decrease in intra-abdominal pressure ≥5 mmHg from baseline (before the procedure), and a decrease in episodes of fever or resolution of fever. (Clinical signs and symptoms like vomiting, abdominal pain, and feed acceptability cannot be assessed adequately in mechanically ventilated patients. Therefore, parameters like vasopressor requirement and intra-abdominal pressure, which are routinely observed in these patients, were assessed to determine clinical success.)

Clinical success was defined by the significant reduction in all the PFCs, along with the resolution of symptoms and the removal of the percutaneous drains. Technical success was defined as the successful insertion of the transgastric drain through both gastric walls on follow-up CT.

Internalization of transgastric drain

Once the collection size was significantly reduced but continued to drain pancreatic fluid, the transgastric drain was internalized.

**Figure 2:**
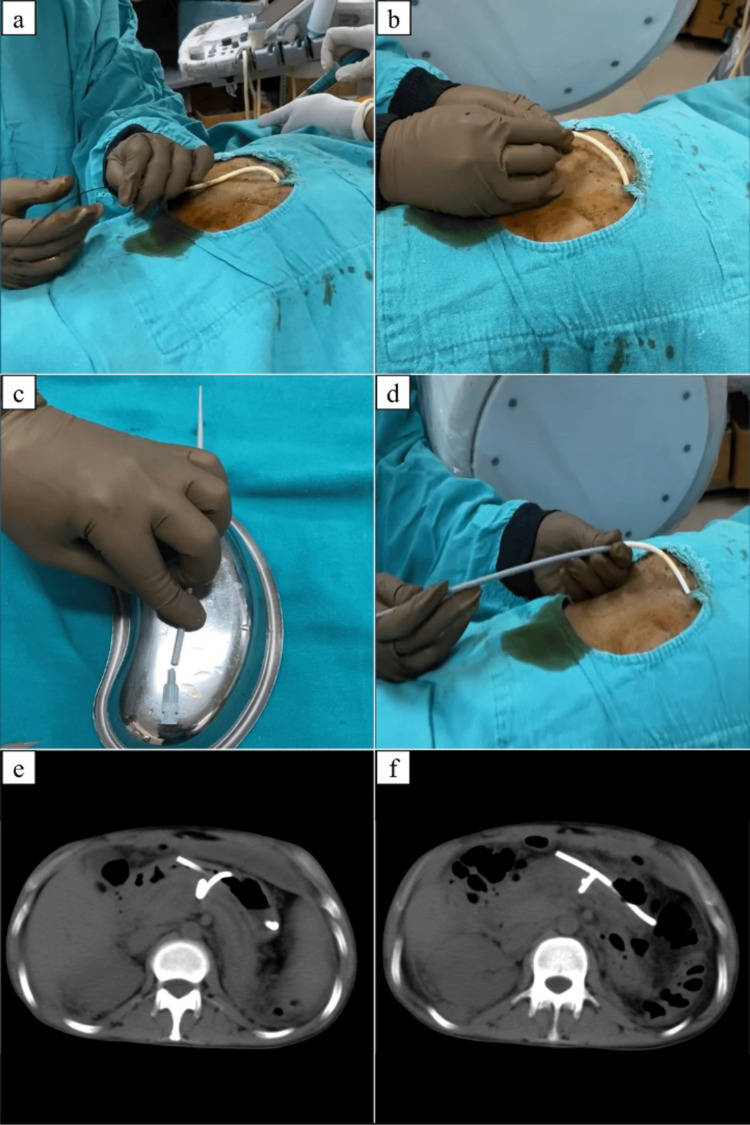
Steps of internalization of transgastric drain The stomach is inflated with air before the procedure. (a) Terumo guidewire was inserted through the drain into the collection. (b) One end of the drain and its two wires are cut, while the Terumo guidewire is still in place and the cut end is removed. (c) One end of Coon’s dilator is cut. (d) The cut end of Coon’s dilator is used to push the drain inside the stomach. (e and f) CT images showing the final position of the internalized drain within the stomach after the removal of the guidewire and dilator

As shown in Figure 2, the stomach was inflated by orally administering an effervescent that contains citric acid, sodium bicarbonate, and sodium carbonate. The locking loop catheter was detached from the urobag and cleaned thoroughly with betadine. After painting and draping, the sutures were cut. Under fluoroscopic guidance, an angled 0.035 guidewire (Terumo Corporation, Tokyo, Japan) was passed through the catheter so that the wire crossed the tip of the catheter. The external part of the catheter was cut along with its two small wires and removed with the guidewire in situ. The back (hub) end of 10 Fr Coon's dilator was cut and passed through the guidewire. The remaining part of the catheter was pushed using the back end of this dilator under continuous fluoroscopic guidance. Once the drain was completely within the stomach lumen, the guidewire and dilator were removed.

Data was compiled using Microsoft Excel (Microsoft Corporation, Redmond, WA, USA), and analysis was done using SPSS Statistics version 23 (IBM Corp. Released 2015. IBM SPSS Statistics for Windows, Version 23.0. Armonk, NY: IBM Corp.). Continuous variables were expressed as mean and standard deviation, whereas categorical variables were represented as frequency and proportion.

## Results

Demographics

Male patients (n=37, 84%) outnumbered female patients. The mean age of the study sample was 31.9±14.1 years. Out of 44 patients, 21 (47.7%) had biliary pancreatitis, and 16 (36.3%) were due to alcohol intake.

Imaging parameters related to PFC

Walled-off necrosis requiring transgastric PCD was present in 44 patients, 32 of whom were infected. The median size of the fluid collection (WOPN) was 250 cc. The minimum size was 20 cc, and the maximum size was 3000 cc. The most frequent location of the collection was the lesser sac, which was seen in 100% of study participants, followed by the left pararenal space (86.4%) and the left paracolic gutter (63.6%). The size of the transgastric drain (locking loop catheter) used was 10 Fr in 21 (47.7%) patients and 12 Fr in 23 (52.3%) patients. CT guidance was used for transgastric drain placement in 34 (77.3%) patients, and USG-guided transgastric drains were placed in 10 (22.7%) patients.

Technical success

Technical success in drain placement was achieved in 41 (93%) out of 44 patients. In three (5%) patients, paragastric drains were placed inadvertently. In one out of these three patients, the paragastric drain was later replaced by another transgastric drain, which was later internalized. In the rest of the two patients, the paragastric drain was kept in situ until the resolution of collection and then directly removed.

Internalization of transgastric drain in the WOPN group

Out of 44 patients with WOPN, internalization of the transgastric drain was attempted in 12 patients. Out of 12 patients, successful internalization was possible in 11 patients. In one patient, internalization failed, and both ends of the drain got displaced in the stomach during the process, which was removed endoscopically. In 32 patients, internalization was not attempted. Two out of these 32 patients had paragastric drains that could not be internalized and were subsequently removed. The rest of the 30 patients had transgastric drains but were removed directly after the resolution of collection and a gradual decrease in the drain output. Thus, the total number of patients where the transgastric drain was successfully internalized was 11 (25%), and where internalization was not done was 33 (75%).

Internalized Transgastric Drain Group (n=11)

In nine (81.8%) patients, additional percutaneous drains were placed for drainage. The median number of extra drains was 2 (range 1-3). There was a need for endoscopic procedures in 10 (90.9%) patients. None of the patients required surgical procedures. Clinical success was achieved in all 11 patients (100%). None of the patients died during the disease course (Table 1).

**Table 1:** Outcome analysis among WOPN patients where transgastric drain was internalized vs where the drain was not internalized * Z-test for proportions, θ Mann-Whitney test, p<0.05 significant ENPD: endoscopic nasopancreatic drainage, IQR: interquartile range, PCD: percutaneous drainage, PD: pancreatic duct, VARD: video-assisted retroperitoneal debridement, WOPN: walled-off pancreatic necrosis

	Transgastric drain not internalized (n=33)	Transgastric drain internalized (n=11)	p-value
Reinterventions in case of displaced drain or re-accumulation of collection	4 (12.1%)	1 (9.1%)	0.741*
Need for other percutaneous drains	28 (84.8%)	9 (81.8%)	0.872*
Number of other drains			
Median (IQR), (range)	2 (1-3), (1-5)	2 (1-2), (1-3)	0.575 ^θ^
Endoscopic procedures	3 (9%)	3 (27.3%)	0.128*
Nagi stent	0	0	
ENPD	0	1 (33.3%)	
PD stenting	1 (3%)	0	
ENPD + PD	0	0	
Cystogastrostomy	2 (6%)	1 (33.3%)	
Endoscopic papillotomy	0	1 (33.3%)	
Surgical procedures	10 (30.3%)	0	0.037
Laparotomy with bowel repair	1 (3%)	0	
VARD	4 (12%)	0	
Necrosectomy	4 (12%)	0	
Necrosectomy with bowel repair	1 (3%)	0	
Clinical success	21 (65.6%)	11 (100%)	0.018*
Adverse events (overall)	8 (24.2%)	1 (9.1%)	0.242*
Death	10 (30.3%)	0	0.037
Time of first drain placement from date of onset of pancreatitis in days median (IQR), (range)	32 (24-47), (14-93)	45 (27 - 104), (21-150)	0.203 ^θ^
Duration of hospital stay in days			
Total duration median (IQR), (range)	55 (40-88), (11-116)	57 (34-71), (23-80)	0.487 ^θ^
From the time of placement of first PCD till discharge median (IQR), (range)	42 (28-70), (3-101)	37 (17-55), (13-69)	0.284 ^θ^
Duration of PCD placement			0.010 ^θ^
Median (IQR), (range)	97 (62-146), (30-245)	42 (37-70), (24-210)	

Non-internalized Transgastric Drain Group (n=33)

There was a need for other percutaneous drains in 28 (84.8%) patients. The median number of extra drains was 2 (range 1-5). There was also a need for other procedures (endoscopic, surgical, or both) in 15 (34%) patients. Endoscopic procedures included a metallic stent (NAGI stent, Taewoong Medical Co. Ltd., South Korea) in one (3%), pancreatic stenting in one (3%) individual, and a cystogastrostomy (double pigtail stent) in two (6%) patients. Surgical procedures were required in 10 (30.3%) patients. Clinical success was achieved in 21 out of 33 patients (65.6%). One patient had a post-surgery gastroduodenal artery bleed, which was managed by endovascular embolization. In another patient, there was bleeding from the left lumbar drain post-surgery, which was managed conservatively. In one patient, there was a hemorrhage within the collection near the transgastric drain, but no arterial source was identified, and the patient was managed conservatively. Ten (30.3%) patients died during the course of the disease. Two (6%) patients could not be followed up as they requested discharge against medical advice (Table 1). Reintervention was required in four (12.1%) patients. In all four patients, transgastric drains were reinserted because of the accidental removal of the previous drain and later removed after the resolution of collection.

Adverse events were seen in eight (25.8%) patients in which the transgastric drain was not internalized, as compared to only one (9.1%) patient in which the transgastric drain was internalized. Adverse events were not necessarily related to the placement of transgastric PCD. Bleeding due to the transgastric drain itself was documented in one patient, which was resolved with conservative management. However, the majority of explorations were due to the worsening of sepsis (eight out of 10 in the first group) and one each for enteric leak and bleeding.

Time of drain placement, total duration of drain placement, and duration of hospital stay in internalized and non-internalized groups

The median time of first drain placement from the date of onset of pancreatitis was lower in patients where the transgastric drain was not internalized (32 days, range 14-193) compared to patients where the transgastric drain was internalized (45 days, range 21-150). The median duration of hospital stay in patients in whom the transgastric drain was not internalized was 55 days (range 11-116) in comparison to 57 days (range 23-80) in whom the transgastric drain was internalized. The median duration of hospital stay from the time of placement of the first PCD until discharge was higher in patients where the transgastric drain was not internalized (42 days, range 3-101) as compared to patients where the transgastric drain was internalized (37 days, range 13-69). The median duration of all PCD placed was significantly higher (p=0.010) in patients where the transgastric drain was not internalized (97 days, range 30-245) as compared to patients where the transgastric drain was internalized (42 days, range 24-210).

## Discussion

Management of pancreatitis-related fluid collection has changed over time. Initially, surgery was the only preferred method, but in the 1980s, with the advancements in image-guided procedures, PCD gained popularity. Subsequently, with the advent of endoscopy, various methods of endoscopic drainage became an integral part of non-surgical methods of pancreatic fluid drainage.

Role of transgastric PCD in the management of pancreatic walled-off necrosis

In the past, open necrosectomy was regarded as the gold standard in the care of necrotic pancreatic collections. However, minimally invasive approaches were investigated to avoid surgery and prevent surgical morbidity. PCD alone, without any surgical procedure, has a success rate of 35%­ to 84%, with mortality of 5.6% to 34% and morbidity of 11% to 42% [2,3]. The morbidity in the majority was associated with pancreatic-cutaneous and pancreatic-enteric fistulas. With the introduction of the "step-up” approach, PCD is now the first line of management, followed by minimally invasive surgical drainage (e.g., VARD) if the patient does not improve clinically. The PANTER trial showed the "step-up" approach over open surgery has a lower percentage of complications, a shorter recovery period, and a lower mortality rate in acute necrotizing pancreatitis [7,8].

The route of PCD depends on the site of fluid collection. For retrogastric PFCs, transabdominal and transgastric routes are used [13]. In the 1980s, multiple authors described the use of the transgastric route for the drainage of retrogastric pancreatic pseudocysts. Matzinger et al. (1988) placed transgastric drains in 12 patients with a clinical success rate of 66% (n=8) and no adverse complications [14]. vanSonnenberg et al. (1989) documented a 100% success rate in eight patients with transgastric drain placement, with one requiring an additional surgical gastrostomy [15]. Sacks et al., Andersson et al., and Curry et al. placed a double J stent percutaneously with one end in the pseudocyst and another end within the stomach, creating a percutaneous cystogastrostomy [16-18]. This helped in the internal drainage of cyst content into the stomach and avoided the formation of a cutaneous fistula [17]. All of these studies showed 100% clinical success rates.

Even though the benefits of transgastric PCD for the management of pseudocysts are well documented in the literature, its specific role in cases of WOPN is not clearly defined. Rey et al. (2022) compared the outcomes of transabdominal vs. transgastric drainage in 18 cases of acute peripancreatic collections where six patients had walled-off necrosis. Transgastric PCD was placed in nine patients, and successful drainage was seen in all cases. None of the patients with transgastric PCD required surgical drainage [19].

In our study, 44 patients with walled-off necrosis were enrolled in whom transgastric PCD placement was attempted. Transgastric PCD was technically feasible in 41 patients (93%). In three (5%) patients, subgastric drains were placed inadvertently. In one of these three patients, the paragastric drain was replaced by another transgastric drain, which was later internalized. In the rest of the two patients, the paragastric drain was kept in situ until the resolution of collection and then directly removed. Ten (22.7%) patients required additional surgical procedures, and six (13.6%) required additional endoscopic procedures. Clinical success was achieved in 32 (72.7%) patients.

One of the major concerns in the management of pancreatitis-related fluid collections is pancreatic duct disruption. Duct disruption can lead to the re-accumulation of fluid, the persistence of symptoms after the drain is removed, and a prolonged hospital stay. Endoscopic pancreatic duct stenting is the preferred minimally invasive treatment in the management of pancreatic duct disruption, as it helps in the internal drainage of fluid collection [20]. However, stenting the pancreatic duct and bridging the leaking duct is technically challenging. In such cases, other forms of internal drainage, like cystogastrostomy, can be done using minimally invasive techniques (percutaneously and endoscopically). The transgastric drain can also be used to create a cystogastrostomy, as explained in our study. Andersson et al. (2002) and Canty and Weinman (2001) also recommended the internalization of drains for the same reason [17,21].

As in the case of a pseudocyst, managing pancreatic duct disruption in cases of WOPN can be done with the help of internal drainage (percutaneously or endoscopically). A recent study has suggested placing at least one transgastric drain in cases of necrotic pancreatic collection, which helps in the internal drainage of contents, especially in cases of PD disruption [21].

In our study, internalization of the transgastric drain was done in 11 out of 44 patients. Higher clinical success (11 (100%)) was seen in patients where internalization of the transgastric drain was achieved (Table 1). None of the patients died in the internalization group, compared to 10 (30.3%) deaths in the non-internalization group. This may be due to the fact that patients in the latter group were sicker and had multiple collections, as 10 out of 33 (30.3%) required surgical intervention.

The time of first drain placement from the date of onset of pancreatitis was lower in patients where the transgastric drain was not internalized (median 32 days) compared to the non-internalized patient group (median 45 days). The median duration of PCD placement, from the time of insertion of the first PCD till the removal of the last PCD/internalization of transgastric PCD, was significantly lower in patients where transgastric drain was internalized (42 days) as compared to patients where internalization was not done (97 days) (p=0.010).

In two patients, instead of transgastric drain internalization, endoscopic internal drainage (PD stenting) was done. Internalization can reduce the time to percutaneous catheter removal in cases with WOPN, which in turn reduces the morbidity and decreases the need for additional interventions (percutaneous or endoscopic) or surgery.

Role of transgastric as a flushing catheter in WOPN

Many studies have shown that the use of more than one drain increases the success rate, as lavage of the cavity is possible with saline to remove the debris and necrotic tissue [22,23]. In our study as well, 36 (81.8%) patients with walled-off necrosis required additional percutaneous drains for the drainage of multiple collections. In 21 (58.3%) patients, a percutaneous drain was placed in the peripancreatic or pararenal space, which was in continuity with the lesser sac collection where transgastric PCD was placed. In these cases, transgastric PCD was used to flush saline into the collection, which was simultaneously aspirated through the larger PCD placed in the peripancreatic or pararenal space.

Complications associated with transgastric drainage

Displacement of the percutaneous cystogastrostomy stent outside the collection or into the stomach, gastric wall hematoma, and tube blockage are some of the known complications, as shown in studies by vanSonnenberg et al., Sacks and Robinson, Henriksen and Hancke, and Grosso et al. [15,16,24,25]. In our study, the transgastric drain was displaced and reinserted in four patients. During internalization, in three patients, the drain was dislodged into the stomach and removed using endoscopic guidance. In the successfully internalized group, the internalized catheter got dislodged into the stomach accidentally in two patients. These displaced drains were removed endoscopically. However, no significant collection was seen, and no further drainage procedures were done.

Another common complication seen during transgastric drain placement is inadvertent subgastric drain placement, which usually occurs when the stomach is not well distended. Due to the elasticity of the stomach wall, it becomes difficult to puncture. A subgastric drain was placed in two patients with WOPN. The disadvantage of these drains is that they cannot be internalized. Thus, to prevent subgastric placement of the drains, proper distension of the stomach should be done before puncturing its anterior wall.

Pancreatic-cutaneous fistulas are one of the most common complications of PCD. van Baal et al. (2011), in a systematic review of the role of PCD in infected pancreatic necrosis, documented the development of pancreatic-cutaneous fistula as the most common complication (51.5%) related to PCD [26]. In our study, none of the patients developed pancreatic-cutaneous fistulas at the transgastric drain site. This helped in retaining the drain for a longer period until the complete resolution of collection. This possible explanation may be the absence of main duct involvement in patients not requiring internalization of the PCD.

Limitations

In our study, only patients with a transgastric drain in situ were included. We did not have a control group where a transabdominal percutaneous drain or endoscopic cystogastrostomy stent was placed for comparison.

## Conclusions

Transgastric drain placement and successful internalization in any form help in the early resolution of peripancreatic and abdominal collections. It also reduces the time to percutaneous catheter removal, which in turn reduces the morbidity and decreases the need for additional interventions or surgery.
